# M‐type K^+^ channels in peripheral nociceptive pathways

**DOI:** 10.1111/bph.13978

**Published:** 2017-09-17

**Authors:** Xiaona Du, Haixia Gao, David Jaffe, Hailin Zhang, Nikita Gamper

**Affiliations:** ^1^ Department of Pharmacology, The Key Laboratory of Neural and Vascular Biology, Ministry of Education Hebei Medical University Shijiazhuang China; ^2^ The Key Laboratory of New Drug Pharmacology and Toxicology Shijiazhuang Hebei Province China; ^3^ School of Biomedical Sciences, Faculty of Biological Sciences University of Leeds Leeds UK; ^4^ Department of Biology, UTSA Neurosciences Institute University of Texas at San Antonio San Antonio TX USA

## Abstract

Pathological pain is a hyperexcitability disorder. Since the excitability of a neuron is set and controlled by a complement of ion channels it expresses, in order to understand and treat pain, we need to develop a mechanistic insight into the key ion channels controlling excitability within the mammalian pain pathways and how these ion channels are regulated and modulated in various physiological and pathophysiological settings. In this review, we will discuss the emerging data on the expression in pain pathways, functional role and modulation of a family of voltage‐gated K^+^ channels called ‘M channels’ (KCNQ, K_v_7). M channels are increasingly recognized as important players in controlling pain signalling, especially within the peripheral somatosensory system. We will also discuss the therapeutic potential of M channels as analgesic drug targets.

**Linked Articles:**

This article is part of a themed section on Recent Advances in Targeting Ion Channels to Treat Chronic Pain. To view the other articles in this section visit http://onlinelibrary.wiley.com/doi/10.1111/bph.v175.12/issuetoc/

**Abbreviations**
BBB: blood–brain barrier
BNB: blood‐nerve barrier
DH: dorsal horn
DRG: dorsal root ganglion
G9a: euchromatic histone‐lysine N‐methyltransferase 2
PSNL: partial sciatic nerve ligation
REST: repressor element 1‐silencing transcription factor
SAR: structure–activity relationship
SIN3A: SIN3 transcription regulator family member A
TRPV1: transient receptor potential cation channel subfamily V member 1
WDR: wide dynamic range
XE991: 10,10‐bis(4‐Pyridinylmethyl)‐9(10H)‐anthracenone dihydrochloride

## Introduction

Pathological pain is a vast and unmet clinical problem, which brings about poor quality of life for sufferers and puts a colossal burden on healthcare systems worldwide. In European countries, national annual economic costs of chronic pain run into billions and amounts to 3–10% of gross domestic products (Breivik *et al.,* 2013). The available statistics suggest that in the United States, total incremental costs of health care due to pain reaches up to hundreds of billions annually, which is higher than the combined costs of cancer and diabetes (Anonymous, 2016). It is clear that other parts of the world are affected to a similar degree, so pain is indeed a worldwide health, societal and economic concern. Yet, despite years of research and investment, there is no ultimate clinical solution to pathological pain and opioids, known to humanity since ancient times, are still a gold standard. While in recent decades there has been a dramatic progress in our understanding of the molecular and cellular mechanisms of pain, the rational design of new therapies based on this mounting knowledge is painstakingly slow. Part of the problem is that most targets for current analgesics are within the CNS and, thus, often cause cognitive and behavioural side effects and are subject to tolerance and addiction issues. Current strategies for peripheral analgesia involve the local application of fairly non‐specific drugs (such as the non‐specific http://www.guidetopharmacology.org/GRAC/FamilyDisplayForward?familyId=82 blockers, e.g. http://www.guidetopharmacology.org/GRAC/LigandDisplayForward?ligandId=2623) or drugs that are specific for broadly expressed targets [e.g. http://www.guidetopharmacology.org/GRAC/LigandDisplayForward?ligandId=2536, a conotoxin inhibitor of N‐type http://www.guidetopharmacology.org/GRAC/FamilyDisplayForward?familyId=80 (Ca_v_2.2 channels)]. These strategies can be robust but often result in the complete loss of sensory and motor fibre activity. Drug discovery for new analgesics mostly focuses on targets that are ‘specific’ for nociceptive neurons, but this has also proven difficult due to either widespread expression patterns [such as, for example with http://www.guidetopharmacology.org/GRAC/ObjectDisplayForward?objectId=507 (Lee *et al.,* 2015)] or the existence of closely related isoforms in other tissues (such as with the voltage‐gated Na^+^ channels; Wood, 2013). Therefore, new ideas for therapies and new molecular targets for these therapies are urgently needed.

Here, we review the current literature on the expression in pain pathways, functional role and a potential for therapeutic targeting of a family of K^+^ channels called ‘M channels’ (http://www.guidetopharmacology.org/GRAC/ObjectDisplayForward?objectId=560&familyId=81&familyType=IC which are increasingly recognized as one of the important mechanisms controlling nociceptive fibre activity. Several earlier reviews on this or related topics are available (Munro and Dalby‐Brown, 2007; Gribkoff, 2008; Rivera‐Arconada *et al.,* 2009; Wickenden and McNaughton‐Smith, 2009; Du and Gamper, 2013; Wang and Li, 2016), so here, we will focus on recent developments in the field as well as on the emerging understanding of the sites of analgesic efficacy of M channel potentiating drugs (‘openers’) within the mammalian nervous system. Since most of the available evidence concerns the M channels expressed within the peripheral somatosensory system, we will mainly focus on the role of M channels in peripheral pain pathways.

## M channels: fit to control

**Figure 1:**
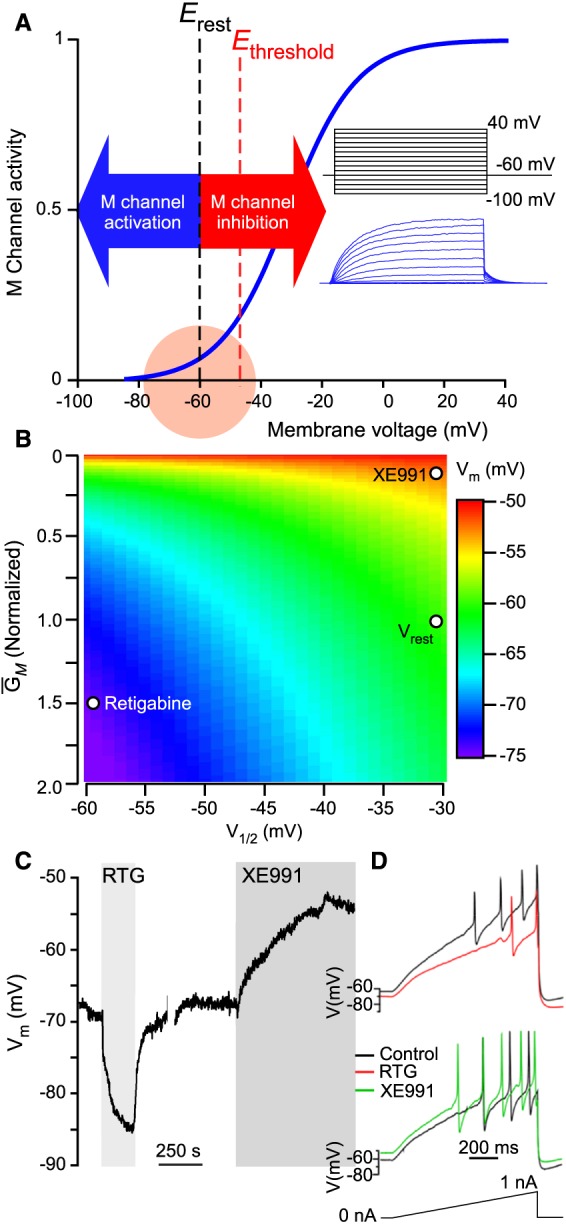
Role of M channels in sensory neurons from a biophysical perspective. (A) Schematized voltage‐dependence of an M channel (Kv7.2/Kv7.3) with relation to the resting membrane potential and firing threshold of a generalized C‐fibre nociceptor. Inset shows M currents recorded in a CHO cell, overexpressing K_v_7.2 and K_v_7.3, in response to the voltage steps depicted in the diagram above the traces. (B) Simulation of the effect of modulation of M channel (K_v_7.2/K_v_7.3) activity with an ‘opener’, retigabine and blocker, XE991, on steady‐state membrane potential. Steady‐state membrane potential (Vm) arises when net current across the plasma membrane is zero (in this example, for only ‘M’ and leak currents). Varying M channel voltage‐dependence and maximum conductance (G_*M*_) shifts V_m_ (leak currents were kept constant). Parameters for V_rest_, retigabine and XE991 were obtained from Linley *et al*. (2012b). (C) Current‐clamp recording of the effect of retigabine and XE991 on the V_m_ recorded from a cultured small‐diameter rat nociceptor in current‐clamp mode (modified from Du *et al*., 2014, with permission). (D) Effects of retigabine and XE991 on the firing threshold and frequency of a cultured small‐diameter mouse nociceptor; stimulus ramp is shown below the traces.

M channels (K_v_7.1–K_v_7.5 coded for by KCNQ1‐KCNQ5 genes) are voltage‐gated K^+^ channels with interesting biophysical properties affecting neuronal excitability (Figure 1; Delmas and Brown, 2005; Gamper and Shapiro, 2015). In particular, they have a very negative threshold for activation, do not inactivate and have slow activation and deactivation kinetics. If the density of K_v_7 channels is sufficiently large (relative to the density of ‘leak’ channels, or other channels that open around the resting potential), they may contribute to the resting potential of a neuron (Delmas and Brown, 2005; Huang and Trussell, 2011; Du *et al.,* 2014). As a result, M channel inhibition causes depolarization, a reduction in the threshold current and rheobase and an increase in input resistance, while their activation hyperpolarizes neuronal plasma membrane and reduces input resistance, making the neuron more resistant to firing action potentials (Figure 1; Gamper and Shapiro, 2015). Numerous reports indeed confirm that acute K_v_7 channel inhibition leads to increased excitability of various types of central and peripheral neurons while loss‐of‐function mutations in KCNQ genes results in debilitating excitability disorders such as epilepsy, deafness and cardiac arrhythmia (reviewed in Jentsch, 2000; Delmas and Brown, 2005; Gamper and Shapiro, 2015).

M channels in neurons are inhibited by stimulation of http://www.guidetopharmacology.org/GRAC/FamilyDisplayForward?familyId=2 (M receptors) and, therefore, serve as a mechanism for the excitatory action of the neurotransmitter http://www.guidetopharmacology.org/GRAC/LigandDisplayForward?ligandId=294 (Brown and Adams, 1980). By now, it is firmly established that http://www.guidetopharmacology.org/GRAC/ObjectDisplayForward?objectId=13, http://www.guidetopharmacology.org/GRAC/ObjectDisplayForward?objectId=15 and http://www.guidetopharmacology.org/GRAC/ObjectDisplayForward?objectId=17 receptors, as well as many other GPCRs of a similar type (e.g. http://www.guidetopharmacology.org/GRAC/ObjectDisplayForward?objectId=42&familyId=10&familyType=GPCR, purinergic http://www.guidetopharmacology.org/GRAC/FamilyDisplayForward?familyId=52 and angiotensin II http://www.guidetopharmacology.org/GRAC/FamilyDisplayForward?familyId=6 receptors), inhibit M channels (Shapiro *et al.,* 1994; Cruzblanca *et al.,* 1998; Filippov *et al.,* 2006; Zaika *et al.,* 2006, 2007; Linley *et al.,* 2008; Gamper and Shapiro, 2015) and cause excitatory actions in neurons of different types, including the nociceptors (Crozier *et al.,* 2007; Linley *et al.,* 2008; Liu *et al.,* 2010). These GPCRs are coupled to G_q/11_ type of Gα and to PLC. The signalling pathways linking the G_q/11_‐coupled receptor activation with the M channel inhibition have been a subject of intense research over the past few decades and are largely outside the scope of this review (in‐depth reviews on this topic are available; Gamper and Shapiro, 2007, 2015; Hernandez *et al.,* 2008; Hille *et al.,* 2015; Greene and Hoshi, 2016). The major mediators of the inhibition are believed to be (i) the depletion of membrane phosphoinositide http://www.guidetopharmacology.org/GRAC/LigandDisplayForward?ligandId=2387, a factor that is necessary for M channel activity (Suh and Hille, 2002; Zhang *et al.,* 2003; Li *et al.,* 2005); (ii) Ca^2+^ release from the IP_3_‐sensitive Ca^2+^ stores (Cruzblanca *et al.,* 1998; Gamper and Shapiro, 2003; Kosenko and Hoshi, 2013); and (iii) http://www.guidetopharmacology.org/GRAC/FamilyDisplayForward?familyId=286&familyType=ENZYME‐mediated M channel phosphorylation (Hoshi *et al.,* 2003; Zhang *et al.,* 2011b). This GPCR‐mediated M channel suppression is at the core of the role these channels play in the development of persistent inflammatory pain (see below).

## M channel expression in peripheral nociceptive pathways

**Figure 2:**
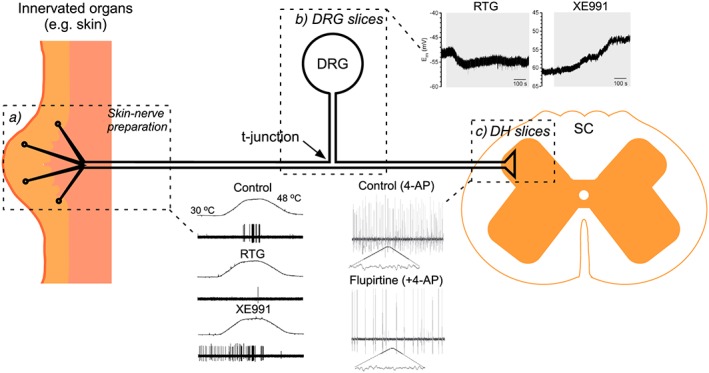
Simplified schematic of a nociceptive neuron. Dotted boxes encircle areas where functional M channel activity has been recorded *in situ* using slices (spinal cord, DRG) or skin‐nerve preparations. Examples of recordings from such preparations are also shown as follows: (a) the effects of retigabine and XE991 on heat‐induced Aδ fibre activity recorded in skin‐saphenous‐nerve preparation (modified from Passmore *et al*., 2012, with permission); (b) the effect of the same compounds on the resting membrane potential of a capsaicin‐sensitive neuron recorded from the DRG ‘slice’ using a sharp electrode recording (modified from Du *et al*., 2014, with permission); (c) the effect of flupirtine on the 4‐AP‐induced excitatory activity recorded extracellularly from substantia gelatinosa in rat spinal cord (modified from Visockis and King, 2013, with permission). Abbreviations: 4‐AP, 4‐aminopiridine; RTG, retigabine, SC, spinal cord.

Peripheral somatosensory neurons are the longest cells in our body (on par with motor neurons) and have a complex, pseudo‐unipolar morphology (schematized in Figure 2). A somatosensory neuron can be subdivided into at least four specialized compartments: (i) a peripheral ending within the innervated tissue (skin, epithelia, muscles, etc.); (ii) a very long axon (fibre); (iii) a central terminal synapsing in the spinal cord; and (iv) a cell body within sensory ganglia. All four compartments have distinct functional roles and distinct anatomical locations (e.g. central terminals are within the CNS while other parts belong to the PNS). Functional M channels are expressed in all these compartments. Thus, dorsal root ganglion (DRG) and trigeminal ganglion (TG) neuron cell bodies generate sizable M currents (Passmore *et al.,* 2003; Rose *et al.,* 2011; King and Scherer, 2012; Zheng *et al.,* 2013; Du *et al.,* 2014), whereas M channel openers reduce and M channel blockers increase somatic excitability of these sensory neurons (Figure 1B, C; Liu *et al.,* 2010; Linley *et al.,* 2012a,b; Du *et al.,* 2014). Functional expression of M channels is confirmed in peripheral sensory axons (Devaux *et al.,* 2004; Rose *et al.,* 2011; Roza *et al.,* 2011; King and Scherer, 2012; Passmore *et al.,* 2012; Peiris *et al.,* 2017), including human sural nerve C fibres (Lang *et al.,* 2008) and human colonic afferents (Peiris *et al.,* 2017). Functional M channels are also reported in nociceptive dorsal roots/central terminals (Rivera‐Arconada and Lopez‐Garcia, 2006) as well as in nociceptive peripheral nerve endings (Linley *et al.,* 2008; Liu *et al.,* 2010; Passmore *et al.,* 2012; Vetter *et al.,* 2013). The relative expression levels and relative functional activity of M channels at these distinct compartments of a peripheral somatosensory neuron are hard to evaluate at present; in case of myelinated fibres, it has been reported that http://www.guidetopharmacology.org/GRAC/ObjectDisplayForward?objectId=561 and http://www.guidetopharmacology.org/GRAC/ObjectDisplayForward?objectId=562 subunits are concentrated at the nodes of Ranvier (Devaux *et al.,* 2004; Roza *et al.,* 2011; King and Scherer, 2012).

The K_v_7 subunit expression profiles in fibres with different sensory modalities are still poorly characterized and even controversial. It is logical to expect that nociceptors, which have intrinsically high firing thresholds, would express more functional M channels as compared to low‐threshold, non‐nociceptive neurons (e.g. low‐threshold mechanoreceptors, proprioceptors, etc.) as the M current is one of the neuronal mechanisms that raises threshold current (see Figure 1); however, this is yet to be confirmed experimentally. The original report describing M channel expression in DRG (Passmore *et al.,* 2003) documented the expression of K_v_7.2, K_v_7.3 and http://www.guidetopharmacology.org/GRAC/ObjectDisplayForward?objectId=564 in nociceptive and non‐nociceptive DRG neurons with K_v_7.2 and K_v_7.3 being predominant subunits. In our studies, we found that K_v_7.2 was indeed more robustly expressed in small‐diameter DRG neurons and non‐myelinated fibres with less abundant (although still detectable) expression in larger neurones (Rose *et al.,* 2011; Huang *et al.,* 2016a). Other studies also reported expression of K_v_7.2 in nociceptive C‐ and Aδ fibres (Wladyka *et al.,* 2008; Passmore *et al.,* 2012); in addition, the expression of K_v_7.5 was found in larger, myelinated fibres (Wladyka *et al.,* 2008). However King and Scherer (2012) came to the opposite conclusion with regard to K_v_7.2 and K_v_7.5 expression: they found that K_v_7.2 is expressed at higher levels in larger, presumably non‐nociceptive, neurons while small‐diameter DRG neuron cell bodies and C fibres expressed lower levels of K_v_7.2 but high levels of K_v_7.5.

The pharmacological profile of M currents recorded from cultured nociceptive DRG neurons speaks against a strong contribution of K_v_7.5, since native M currents in DRG neurons display reasonable sensitivity to http://www.guidetopharmacology.org/GRAC/LigandDisplayForward?ligandId=2596 (Passmore *et al.,* 2003, 2012; Linley *et al.,* 2008, 2012a; Rose *et al.,* 2011), as do the heterologousely overexpressed K_v_7.2 and K_v_7.3 channels, while K_v_7.5 has a weaker sensitivity to this blocker (Schroeder *et al.,* 2000). This, however, does not exclude the possibility that K_v_7.5 contribute to some extent, as channels expressed in cultured DRG neurons may not be equivalent to those expressed in native tissue. Future studies will have to resolve this controversy as well as establishing the exact K_v_7 subunit expression profiles in somatosensory fibres of different modalities. This is indeed an important task as it can hold the key to the development of subunit‐specific M channel openers, optimized for targeting channels in peripheral fibres for pain relief (see below).

In addition to the above findings, in another study it was demonstrated that a defined subpopulation of cutaneous mechanoreceptors (rapidly adapting hair follicles and Meissner corpuscles) specifically expresses http://www.guidetopharmacology.org/GRAC/ObjectDisplayForward?objectId=563 (Heidenreich *et al.,* 2012).

## M channels and peripheral fibre excitability *in vitro, in silico* and *in vivo*

There are very clear and undisputed effects of M channel activity on the somatic excitability of cultured DRG and TG neurons (Passmore *et al.,* 2003; Crozier *et al.,* 2007; Linley *et al.,* 2008, 2012a,b; Liu *et al.,* 2010; Mucha *et al.,* 2010; King *et al.,* 2014; Figure 1C). Yet, since cultured sensory neurons are axotomized and are grown in artificial conditions, their physiology may not necessarily accurately represent those of native neurons, since gene expression may be different. Therefore, a higher level of confidence regarding the functional role of M channels in sensory neuron excitability is gained from acute preparations, such as skin‐nerve preparations and sectioning of acutely dissected ganglia/nerves.

### M channel activity and excitability of spinal nociceptors

Depolarization by the M channel inhibitor, XE991 and hyperpolarization by an M channel activator (‘opener’) http://www.guidetopharmacology.org/GRAC/LigandDisplayForward?ligandId=2601 has been recorded from http://www.guidetopharmacology.org/GRAC/LigandDisplayForward?ligandId=2486‐sensitive neurons using sharp‐electrode recordings from acute DRG slices (Du *et al.,* 2014). In a skin‐saphenous‐nerve preparation, retigabine markedly reduced noxious heat responses of the mechano‐heat Aδ‐ and some C‐fibres (the latter to a somewhat lesser extent), while XE991 increased noxious heat sensitivity and induced ongoing activity at 32°C in Aδ fibres (Passmore *et al.,* 2012). A similar skin‐saphenous‐nerve preparation has been used to demonstrate that M channel inhibition (e.g. by XE991, http://www.guidetopharmacology.org/GRAC/LigandDisplayForward?ligandId=2422 or http://www.guidetopharmacology.org/GRAC/LigandDisplayForward?ligandId=2471) amplified cold transduction by a specific population of C fibre nociceptors: http://www.guidetopharmacology.org/GRAC/ObjectDisplayForward?objectId=500‐expressing C‐mechano‐cold fibres (Vetter *et al.,* 2013). In the same fibres, retigabine inhibited cold‐evoked activity. In another study, the multi‐unit recordings of the lumbar splanchnic nerve in a colon‐nerve preparation demonstrated that the noxious distension‐induced firing was reduced by retigabine and increased by XE991 (Peiris *et al.,* 2017). Collectively, these studies indicate that M channel activity does strongly affect the excitability of acutely isolated spinal Aδ and C fibres.

Although indicative, *in vitro* tests fall short of demonstrating how strongly, if at all, native M channel activity contributes to peripheral nociceptive transmission *in vivo*. Yet there is growing evidence suggesting that this is indeed the case. Thus, plantar hind paw injections of the M channel blocker, XE991, induce moderate pain in rats (Linley *et al.,* 2008; Liu *et al.,* 2010). In accord with this, plantar injections of XE991 increased firing of wide dynamic range (WDR) dorsal horn (DH) neurons in response to both mechanical and thermal stimulation of the hind paw in spinal cord recordings in anaesthetized animals (Passmore *et al.,* 2012). Moreover, hind paw injection of retigabine reduced thermal sensitivity in freely behaving animals (Huang *et al.,* 2016a) and also greatly reduced pain produced by local (hind paw) injections of the inflammatory mediator http://www.guidetopharmacology.org/GRAC/LigandDisplayForward?ligandId=649 (Liu *et al.,* 2010; Huang *et al.,* 2016a).

A recent computational study provided a biophysical foundation to these and similar observations by utilizing a multi‐component model of rat C fibre that included a geometrically realistic nociceptive nerve ending in the skin (Barkai *et al.,* 2017). When sufficiently pronounced, M channel inhibition resulted in ‘spontaneous firing’. Whereas almost complete inhibition was required for spike generation in the soma, a less dramatic decrease (to just below 37%) was required to produce ‘spontaneous’ firing in a nociceptive terminal. The modelling also showed that M current actively prevented excitation of nociceptive terminals in response to a range of subthreshold depolarizations and fairly large (up to 15 mV) ‘spontaneous’ fluctuations of resting membrane potential. When reduced to 50% of ‘basal’ density, M current still prevented spontaneous firing, but such a reduction dramatically potentiated responses to noxious stimulation (e.g. with simulated ‘capsaicin’).

### M channel activity and excitability of trigeminal nociceptors

K_v_7.2 immunoreactivity (Abd‐Elsayed *et al.,* 2015) and sizable M currents were reported in small‐diameter TG neurons in culture (Linley *et al*., 2012a, b; Ooi *et al.,* 2013; Abd‐Elsayed *et al.,* 2015) and in the whole‐cell patch clamp recordings from acute whole‐mount trigeminal ganglia (Kanda and Gu, 2017). These studies showed that, similar to spinal neurons, M channels are involved in setting resting membrane potential and rheobase in cold‐sensing TG nociceptors (Abd‐Elsayed *et al.,* 2015) and also in most neurons that are sensitive to cold but are not primarily cold‐sensing (Kanda and Gu, 2017). Interestingly, it was also reported that inhibition of M channel activity by cold contributes to cold sensitivity of the latter type of neurons. Retigabine (i.p.) alleviated cold hyperalgesia in two orofacial neuropathic pain models (Abd‐Elsayed *et al.,* 2015), further supporting the active role M channels play in the control of trigeminal nociceptor excitability *in vivo*. In addition to the above studies, Linley *et al*. (2012a) analysed the unexpected potentiation of M channel activity by http://www.guidetopharmacology.org/GRAC/LigandDisplayForward?ligandId=2098 in TG and DRG neurons and found that, mechanistically, the modulation was similar in both types of sensory neurons.

### M channels in nociceptive mechanotransduction

The studies discussed above established a role for M channels in modulating the sensitivity of peripheral nociceptive fibres to heat and cold. However, the role of M channels in mechanical sensitivity is less certain. In skin‐saphenous‐nerve preparations from naïve CD1 mice, retigabine had no significant effect on firing in response to mechanical stimulation of either slowly or rapidly adapting mechano‐sensitive Aδ and C fibres, while two out of four rapidly adapting Aδ fibres responded to XE991 with increased firing rate (Roza and Lopez‐Garcia, 2008). However, in the same study, retigabine strongly suppressed firing in axotomized mechanically‐sensitive fibres of either type. Mice with genetic deletion of *Kcnq2* in cells derived from the neural crest (including DRG neurons) displayed both thermal and mechanical hyperalgesia (King *et al.,* 2014). Consistently, in another study, mice with global genetic deletion of K_v_7.3 (*Kcnq3* ^−/−^) and down‐regulation of K_v_7.2 (*Kcnq2* ^+/−^) displayed increased firing rates of mechanosenstive D‐hairs (Aδ‐low‐threshold mechanoreceptors) in response to mechanical stimulation (Schutze *et al.,* 2016). Yet the effect was modest and no obvious behavioural phenotype was reported (although this could have been due to only partial loss of K_v_7.2). Finally, it was also reported that peripheral nerve endings of rapidly adapting cutaneous hair follicles and Meissner corpuscle mechanoreceptors from mice and humans abundantly express K_v_7.4 (Heidenreich *et al.,* 2012). Moreover, mice with genetic deletion of *Kcnq4* displayed increased sensitivity of rapidly adapting mechanoreceptors to low‐frequency vibration. In accord with these findings, humans with DFNA2 (a slowly progressing, autosomal dominant form of hearing loss) due to loss‐of‐function mutations in *KCNQ4* were better than age‐matched controls at detecting low frequency skin vibration (Heidenreich *et al.,* 2012). Altogether, these data suggest that the contribution of K_v_7 subunits to the modulation of mechanical sensitivity varies in different types of mechanoreceptors; however, elucidation of the exact role of M channels in the mechanosensitivity of peripheral afferents (and how it might change in pathological pain states) requires further research.

## M channel suppression as a general mechanism for peripheral nerve hyperexcitability

The findings discussed above established that (i) M channels control the excitability of neurons that express them at sufficient levels; (ii) loss of M channel function results in an overexcitable neuron; and (iii) at least some peripheral nociceptive fibres express sufficient levels of functional K_v_7 subunits for these to influence fibre excitability. The most logical projection from these conclusions is that inhibition, down‐regulation or any other form of loss of M channel function in nociceptive fibres might result in increased excitability, nociception and pain. On the other hand, peripheral M channel activation could be analgesic. Below, we will consider growing experimental evidence supporting the above considerations.

### Inflammatory pain

As mentioned earlier, many G_q/11_‐coupled GPCRs were shown to acutely and potently inhibit M channel activity. Tissue inflammation brings about a specific environment, in which inflamed/damaged tissue as well as cells involved in the immune response release various chemical factors (inflammatory mediators; e.g. prostaglandins, leukotrienes, interleukins, bradykinin, substance P, http://www.guidetopharmacology.org/GRAC/LigandDisplayForward?ligandId=1713, growth factors, proteases, protons and http://www.guidetopharmacology.org/GRAC/LigandDisplayForward?ligandId=2509) in order to orchestrate defensive and reparative processes or as a result of the loss of tissue integrity (Serhan and Savill, 2005; Ferrero‐Miliani *et al.,* 2007). These same factors often ‘sensitize’ or even directly excite nociceptive afferents triggering inflammatory pain and hyperalgesia. Many of the receptors of these inflammatory mediators are GPCRs that are coupled to the G_q/11_‐PLC signalling pathway (similar to muscarinic M_1_ receptors). These include B_2_ receptors (Couture *et al.,* 2001; Petho and Reeh, 2012), http://www.guidetopharmacology.org/GRAC/ObjectDisplayForward?objectId=262 receptors (Kuhn *et al.,* 1996), http://www.guidetopharmacology.org/GRAC/ObjectDisplayForward?objectId=348 (PAR2; Bushell *et al.,* 2006), http://www.guidetopharmacology.org/GRAC/ObjectDisplayForward?objectId=340 (Breyer *et al.,* 1996) and P2Y (Chen and Chen, 1997) receptors (reviewed in Linley *et al.,* 2010). Bradykinin can be considered as one of the prototypic G_q/11_ coupled inflammatory mediators as it is released upon tissue damage and inflammation; its receptors are abundantly expressed in nociceptors; and bradykinin injections or topical applications to wounds/blisters are very painful (Keele, 1967; Dray and Perkins, 1993; Liu *et al.,* 2010; Petho and Reeh, 2012; Linley *et al.,* 2012a). Bradykinin is so active that it has been branded ‘the most potent endogenous algogenic substance ever identified’ (Dray and Perkins, 1993).

Bradykinin is known to inhibit M channels in expression systems and sympathetic and sensory neurons (Cruzblanca *et al.,* 1998; Gamper and Shapiro, 2003; Zhang *et al.,* 2003; Liu *et al.,* 2010; Linley *et al.,* 2012a). Thus, in cultured DRG neurons, bradykinin inhibited M current by 50–60% and increased their excitability, effects that were mimicked by XE991 and antagonized by a retigabine analogue, http://www.guidetopharmacology.org/GRAC/LigandDisplayForward?ligandId=2598 (Liu *et al.,* 2010; Linley *et al.,* 2012a). In accord with these findings, hind paw injections of bradykinin produced prominent pain‐like (‘nocifensive’) behaviour in rats, an effect that can be attenuated by retigabine or mimicked by XE991 (Liu *et al.,* 2010; Zhang *et al.,* 2015; Huang *et al.,* 2016a). Bradykinin was recently also shown to robustly excite colonic afferents, and this effect was also abolished by retigabine and partially mimicked by XE991 (Peiris *et al.,* 2017). It has to be noted that M current inhibition is not the sole excitatory action of bradykinin in DRG neurons; B_2_ receptor stimulation also results in the activation of the excitatory chloride channel, http://www.guidetopharmacology.org/GRAC/FamilyDisplayForward?familyId=130#708 (Liu *et al.,* 2010; Jin *et al.,* 2013), sensitization of TRPV1 (Sugiura *et al.,* 2002) and http://www.guidetopharmacology.org/GRAC/ObjectDisplayForward?objectId=485http://www.guidetopharmacology.org/GRAC/ObjectDisplayForward?objectId=485 (Bandell *et al.,* 2004) channels as well some additional effects, including trafficking of pro‐excitatory channels (Huang *et al.,* 2016b; reviewed in Petho and Reeh, 2012).

Similarly to bradykinin receptors, stimulation of PAR2 (Linley *et al.,* 2008) and http://www.guidetopharmacology.org/GRAC/ObjectDisplayForward?objectId=152 (Crozier *et al.,* 2007) receptors also inhibits M channels in nociceptive DRG neurons, an effect that contributes to the inflammatory pain and itch mediated by these inflammatory GPCRs.

### Neuropathic pain

Neuropathic pain is defined as ‘pain caused by a lesion or disease of the somatosensory nervous system’ (International Association for the Study of Pain, 2017); it is caused by injuries or degeneration within peripheral or central somatosensory pathways. Among chronic pain conditions, neuropathic pain is one of the most debilitating and also one of the most resilient to treatment as it often responds poorly to medication (O'Connor and Dworkin, 2009). Neuropathy is associated with spontaneous and evoked pains that often persist over long periods of time (Campbell and Meyer, 2006). Both peripheral and central mechanisms contribute to the development of neuropathic pain. A state of sustained pathological overexcitability of nociceptive afferents (‘peripheral sensitization’) is considered necessary for the development of most types of neuropathic pain.

When peripheral axons are severed (e.g. by trauma), the nerve stump forms a swelling or endbulb, which then sprouts thin processes, which may re‐connect severed nerve endings, if these are in close enough proximity. If regeneration is obstructed, the endbulb, aborted sprouts and surrounding glia become a bulky tissue formation called a neuroma (Fawcett and Keynes, 1990; Devor, 2006). Both the neuroma (Govrin‐Lippmann and Devor, 1978; Wall and Devor, 1983) and cell bodies of damaged nerves (Wall and Devor, 1983; Amir and Devor, 1997; Liu *et al.,* 2000) generate spurious ectopic firing, which may cause pain on its own but also feeds into the spinal wind‐up mechanisms. The neuropathic deformation of the injured nerve is associated with large‐scale remodelling of the injured and perhaps also neighbouring, uninjured fibres within the affected nerve and the corresponding sensory ganglion. One of the striking features of such remodelling is a marked down‐regulation of several key K^+^ channel genes (including *Kcnq2* and *Kcnq5*) (Rose *et al.,* 2011; King and Scherer, 2012; Du and Gamper, 2013; Cisneros *et al.,* 2015; Laumet *et al.,* 2015). Such a loss of K^+^ channels is expected to result in depolarized and more excitable fibres, which is indeed one of the characteristic of neuropathic afferents and, also, of the DRG neurons from mice with the genetic deletion of K_v_7.2 from sensory neurons (King *et al.,* 2014). The mechanisms underlying the down‐regulation of K^+^ channel genes following nerve injury are under intense scrutiny, and several molecular players have been identified. Thus, we found that a down‐regulation of *Kcnq2* expression triggered by the partial sciatic nerve ligation (PSNL) in rat DRG is dependent on repressor element 1‐silencing transcription factor (REST, also known as NRSF) (Rose *et al.,* 2011). *Kcnq* genes have functional repressor element 1 (NRSE) binding sites (Mucha *et al.,* 2010; Iannotti *et al.,* 2012). Accordingly, viral overexpression of REST in DRG neurons strongly suppressed M current density and increased their tonic excitability (Mucha *et al.,* 2010). REST inhibits transcription by recruiting co‐repressor complexes SIN3A/B and REST corepressor 1; these complexes, in turn, modify target gene regions through chromatin‐modifying enzymes, including histone deacetylases 1/2 (http://www.guidetopharmacology.org/GRAC/FamilyDisplayForward?familyId=8481/2), the histone demethylase http://www.guidetopharmacology.org/GRAC/ObjectDisplayForward?objectId=2669 and the histone methylase http://www.guidetopharmacology.org/GRAC/FamilyDisplayForward?familyId=871#2652 (Ooi and Wood, 2007; Willis *et al.,* 2016). Interestingly, inhibition or genetic deletion of G9a in DRG was shown to abolish the down‐regulation of K_v_7.2 [as well as three other K^+^ channels: http://www.guidetopharmacology.org/GRAC/ObjectDisplayForward?objectId=541, http://www.guidetopharmacology.org/GRAC/ObjectDisplayForward?objectId=553 and http://www.guidetopharmacology.org/GRAC/ObjectDisplayForward?objectId=380 (K_Ca_1.1)] induced by neuropathic injury (Laumet *et al.,* 2015). Inhibition or deletion of G9a also markedly reduced neuropathic hyperalgesia. Notably, baseline REST levels in neurons are low, but neuropathic injury and inflammation triggers REST expression (Mucha *et al.,* 2010; Uchida *et al.,* 2010; Rose *et al.,* 2011; Willis *et al.,* 2016), which, in turn, drives the neuropathic remodelling.

Of note is the fact that M channel expression in neuropathic fibres is reduced, but not abolished (Rose *et al.,* 2011; Cisneros *et al.,* 2015). Accordingly, M channel openers are still efficacious in alleviating neuropathic pain symptoms, presumably by acting on the remaining K_v_7 channels to stabilize the *E*_rest_ and reduce firing in the affected fibres. Thus, retigabine strongly suppressed the spontaneous activity of neuropathic fibres (Bernal *et al.,* 2016), and thermal hyperalgesia produced by PSNL in rats was effectively alleviated by an injection of flupirtine directly into the neuroma (an effect that was reversed by XE991) (Rose *et al.,* 2011). It has to be noted that an increase in K_v_7.2‐positive nodes of Ranvier in nerve‐end neuromas of mice with neuropathic injury (saphenous nerve transection) has also been reported (Roza *et al.,* 2011; Cisneros *et al.,* 2015). Thus, while down‐regulated in the soma and elsewhere, remaining M channels may actually be pooled at the sites of ectopic activity (e.g. at neuroma nerve stubs), as a compensatory mechanism to offset spurious nerve activity. Such an effect would represent an additional mechanism for anti‐nociceptive efficacy of peripherally applied M channel openers.

### Cancer pain

Tumours often trigger pain *via* a combination of inflammatory, neuropathic, ischaemic and tissue compression mechanisms and also by chemical mediators released by cancer cells (Prinsloo *et al.,* 2013). Systemic administration of flupirtine displayed analgesic efficacy in a rat model of prostate bone metastasis (Kolosov *et al.,* 2012), suggesting that a general dampening of excitability within the pain pathways *via* M channel potentiation might have potential as a treatment for cancer pain.

As in the case of neuropathic pain, a marked down‐regulation of K_v_7.2 and K_v_7.3 channel abundance and a reduction of M current amplitudes were reported in DRG neurons in a rat model of bone cancer pain (Zheng *et al.,* 2013); these effects were also accompanied by a hyperexcitable state of the DRG neurons. In a follow‐up study, the same group also showed that the excitability of the C‐fibre‐synapsed WDR neurons in the dorsal horn is also enhanced in the rats developing bone cancer and this effect was also attributed, at least in part, to the M channel suppression (although in the latter study, it was not clear, whether the M current was down‐regulated in the C‐fibre terminals or in the WDR neurons themselves, or at both locations) (Cai *et al.,* 2015). Systemic (Zheng *et al.,* 2013) or intrathecal (Cai *et al.,* 2015) retigabine alleviated mechanical allodynia and thermal hyperalgesia induced by bone cancer and also reduced hyperexcitability of both DRG and WDR DH neurons. Similar to the neuropathic pain models (Rose *et al.,* 2011), despite the down‐regulation of M channel activity/expression, retigabine and flupirtine were still efficacious in alleviating cancer pain even when applied locally (e.g. intrathecally).

## M channel openers

Due to the extensive expression of K_v_7 channels in the nervous and cardiovascular systems and their strong effects on excitability, the therapeutic potential of these channels is not limited to pain but also includes epilepsy, anxiety (Gribkoff, 2008), stroke (i.e. as a protection against excitotoxicity) (Gamper *et al.,* 2006; Bierbower *et al.,* 2015), smooth muscle disorders (Stott *et al.,* 2014) and other diseases. It is therefore not surprising that there is a large drive, both in industry and academia, to identify potent and selective M channel openers. Conveniently, M channels present an unusually rich substrate for pharmacological modulation, and literally thousands of openers acting *via* various distinct mechanisms have already been identified. World Intellectual Property Organization lists well over 2 hundred patents related to M channel openers. Progress in M channel pharmacology has been discussed in several recent reviews (Gribkoff, 2008; Miceli *et al.,* 2008; Du and Gamper, 2013; Grunnet *et al.,* 2014); known mechanisms of potentiation and binding sites for major classes of M channel openers were reviewed by us recently (Du and Gamper, 2013); therefore, here we will just briefly outline the main classes of M channel openers, which display analgesic activity, as well as some major recent developments.

**Figure 3:**
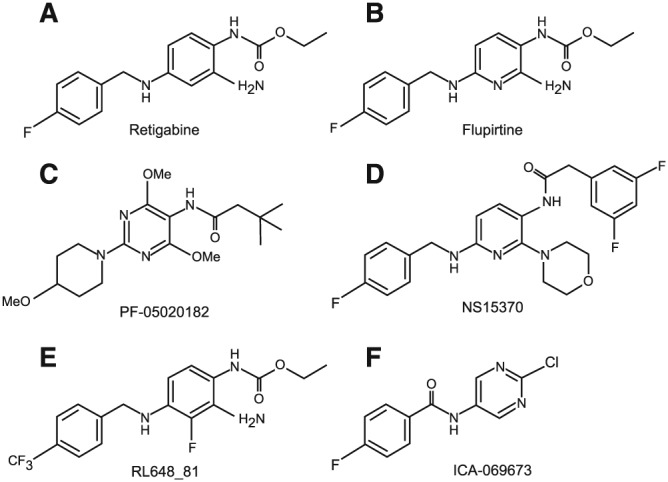
Chemical structures of retigabine and its analogues.

The prototypical M channel openers retigabine (ezogabine) and flupirtine (katadolon, awegal) belong to triaminopyridines (Figure 3A, B); these compounds are potent but relatively non‐selective K_v_7 openers that activate all K_v_7 subunits except of http://www.guidetopharmacology.org/GRAC/ObjectDisplayForward?objectId=560, with K_v_7.3 being the most sensitive subunit (Tatulian *et al.,* 2001; Tatulian and Brown, 2003; Schenzer *et al.,* 2005; Wuttke *et al.,* 2005). Flupirtine has been used clinically in Europe since 1984 as a centrally acting nonopioid analgesic for postoperative, traumatic and cancer pain; it also has muscle‐relaxant activity. The analgesic efficacy of flupirtine is comparable to that of http://www.guidetopharmacology.org/GRAC/LigandDisplayForward?ligandId=2713s such as ibuprofen (Miceli *et al.,* 2008). Although flupirtine was reported to affect http://www.guidetopharmacology.org/GRAC/FamilyDisplayForward?familyId=75 and http://www.guidetopharmacology.org/GRAC/FamilyDisplayForward?familyId=72 (Miceli *et al.,* 2008), little direct evidence exists and it is believed that its analgesic effect is mainly related to its activity as an M channel opener (Devulder, 2010). Retigabine is a clinically approved antiepileptic drug (Stafstrom *et al.,* 2011), but it also consistently displays analgesic efficacy in multiple animal pain models such as temporomandibular joint pain (Xu *et al.,* 2010), visceral pain (Hirano *et al.,* 2007), carrageenan‐induced hyperalgesia (Passmore *et al.,* 2003), trigeminal neuropathy (Abd‐Elsayed *et al.,* 2015), bone cancer pain (Zheng *et al.,* 2013). The clinical use of retigabine has been recently restricted to adjunctive treatment of drug‐resistant partial onset seizures (and only if other treatments have failed or could not be tolerated; Daniluk *et al.,* 2016) due to significant risks of skin discoloration, retinal pigmentation, urinary retention, sedation and QTc prolongation (Daniluk *et al.,* 2016). Nevertheless, retigabine remains a useful research tool and a ‘benchmark’ drug in animal pain models. Its mechanism of action is also fairly well understood. The binding site of retigabine resides within the cytosolic region between S5 and S6 transmembrane domains of K_v_7 channels and contains a critical tryptophan (W236 in K_v_7.2), which is absent in K_v_7.1 (Schenzer *et al.,* 2005; Wuttke *et al.,* 2005). Biophysically triaminopyridines induce a large shift in channel voltage‐dependence towards negative voltages (i.e. retigabine shifts half‐maximal activation voltage of K_v_7.2/K_v_7.3 multimers by more than −30 mV); additionally, these compounds increase macroscopic steady‐state K^+^ conductance at saturating voltages (Tatulian *et al.,* 2001; Tatulian and Brown, 2003; Schenzer *et al.,* 2005; Wuttke *et al.,* 2005; Linley *et al.,* 2012b).

K_v_7 channels are widely expressed within the CNS, PNS and vasculature, yet their subunit distribution is not uniform. While K_v_7.2/K_v_7.3 multimeric channels are believed to dominate the CNS, K_v_7.4 is highly expressed specifically in auditory pathways (as well as in a subpopulation of rapidly adapting cutaneous mechanoreceptors; Heidenreich *et al.,* 2012; see above). K_v_7.1 is mostly expressed in the heart and epithelia (but not in neurons), while vascular smooth muscles more abundantly express K_v_7.5, K_v_7.4 and their multimers (reviewed in Gamper and Shapiro, 2015). Therefore, openers such as retigabine, which discriminate poorly between K_v_7 subunits, are likely to have multiple ‘on‐target’ side effects. Drugs with higher subunit selectivity ‘tailored’ towards specific cell types are needed to achieve more specific pharmacological actions. Indeed, recent efforts in academia and industry have been focused on discovering new drugs or the refinement of existing compounds towards better selectivity and higher potency.

Several groups have performed structure–activity relationship (SAR) analyses of retigabine to produce more potent and selective derivatives. Thus, compounds PF‐05020182 (Figure 3C) (Davoren *et al.,* 2015) and NS15370 (Figure 3D) (Dalby‐Brown *et al.,* 2013), which are ~10 and ~30 fold more potent than retigabine, were synthesized; these are ineffective against K_v_7.1 but do activate K_v_7.4 and K_v_7.5 channels with comparable potency to that of K_v_7.2/K_v_7.3. Thanos Tzounopoulos' group used the SAR approach to generate several improved retigabine derivatives. By introducing a CF3‐group at the 4‐position of the benzylamine moiety and a fluorine at the 3‐position of the aniline ring (Figure 3E), they generated a compound (RL648_81) which is over 15 times more potent than retigabine at K_v_7.2/K_v_7.3 channels (EC_50_ 0.2 μM vs. ~3 μM) and does not affect K_v_7.4 and K_v_7.5 (Kumar *et al.,* 2016). Another recent compound, ICA‐069673 (and related derivatives such as ICA‐27243 and ztz series compounds), displays remarkable selectivity for K_v_7.2 over K_v_7.3 (Figure 3F) (Wickenden *et al.,* 2008; Gao *et al.,* 2010; Wang *et al.,* 2016).

Other M channel openers include acrylamides known as compounds (S)‐1 and (S)‐2 (Wu *et al.,* 2004a,b,c, 2013; L'Heureux *et al.,* 2005) developed by Bristol‐Myers Squibb. Acrylamides share the site of action and specificity profile with retigabine (Bentzen *et al.,* 2006) and display analgesic efficacy in rodent models of migraine (Wu *et al.,* 2004a), inflammatory and neuropathic pain (Wu *et al.,* 2013). A QO series of openers is based on pyrazolo[1,5‐a]pyrimidin‐7(4H)‐one (Jia *et al.,* 2011; Qi *et al.,* 2011; Zhang *et al.,* 2012; Teng *et al.,* 2016); the lead compound, QO‐58 (and QO‐58‐lysine, which has improved bioavailability), has analgesic efficacy in neuropathic and inflammatory pain models (Zhang *et al.,* 2012; Teng *et al.,* 2016). QO‐58 potentiates all K_v_7 channels except K_v_7.3. Benzimidazoles, such as compound B1 (Zhang *et al.,* 2011a; Du and Gamper, 2013), have high selectivity for K_v_7.2 over K_v_7.3, K_v_7.4 and K_v_7.5. Like the QO series, benzimidazoles do not require W236 for their action on K_v_7 channels (reviewed in Du and Gamper (2013). In addition, adamantyl derivatives (Fritch *et al.,* 2010), N‐phenilanthranilic acid derivatives, for example, meclofenamic acid, http://www.guidetopharmacology.org/GRAC/LigandDisplayForward?ligandId=2714 and related compounds (Peretz *et al.,* 2005; Peretz *et al.,* 2007; Brueggemann *et al.,* 2009; Peretz *et al.,* 2010), http://www.guidetopharmacology.org/GRAC/LigandDisplayForward?ligandId=4319 (Zhang *et al.,* 2015) and a Rho kinase inhibitor http://www.guidetopharmacology.org/GRAC/LigandDisplayForward?ligandId=5181 (Zhang *et al.,* 2016) were identified as M channel openers. Of these, fasudil is perhaps unique as it selectively activates K_v_7.4 and K_v_7.4/K_v_7.5 channels but not the other K_v_7 subunits. A zinc coordination complex, http://www.guidetopharmacology.org/GRAC/LigandDisplayForward?ligandId=2597, has also been suggested to display K_v_7 channel opener activity (Xiong *et al.,* 2007), but it appears that the channel potentiation in this case is produced by intracellular zinc, whereas pyrithione acts as a zinc ionophore (Gao *et al.,* 2017).

## Where is the site of analgesic action of M channel openers?

M channel openers display analgesic efficacy when administered peripherally (e.g. plantar paw injections) (Linley *et al.,* 2008; Liu *et al.,* 2010; Huang *et al.,* 2016a), injected into the neuroma site (Rose *et al.,* 2011), focally applied to DRG (Du *et al.,* 2014) or administered intrathecally (Cai *et al.,* 2015) or systemically (reviewed in Du and Gamper (2013). Interestingly, one recent study has demonstrated that even when given orally, the analgesic action of retigabine is almost exclusively peripheral (Hayashi *et al.,* 2014). Thus, most central effects of oral retigabine (anticonvulsant effect, impaired motor coordination, reduced exploratory behaviour) were effectively precipitated by central (i.c.v.) application of XE991. Yet the analgesic effect was not affected. The likelihood therefore is that the main site of analgesic action of retigabine is within the peripheral fibres.

As discussed above, functional M channels have been shown to be present in all major sensory nerve compartments (peripheral and central terminals, soma, axon). Thus, theoretically, M channel activation can suppress the generation, propagation and transmission of nociceptive signals to the CNS.

Experimental data suggest that potentiation of M channel activity at the nociceptive terminals in the skin with retigabine, flupirtine or other openers does indeed reduce basal sensitivity to thermal and mechanical stimuli (Passmore *et al.,* 2012; Vetter *et al.,* 2013; Huang *et al.,* 2016a) and alleviates the nociceptive effects of various peripherally applied algogenic stimuli (Linley *et al.,* 2008; Liu *et al.,* 2010; Passmore *et al.,* 2012; Vetter *et al.,* 2013; Hayashi *et al.,* 2014; Huang *et al.,* 2016a; Peiris *et al.,* 2017). This suggests that nociceptive nerve endings are indeed one of the sites for the analgesic efficacy of the openers.

Intrathecal application of M channel openers also produces an analgesic effect (Cai *et al.,* 2015), suggesting that the transmission of nociceptive signals to the spinal cord can also be controlled by M channels. Yet, in this case, it is hard to pinpoint the site of action exactly, as DRG, dorsal roots, central terminals of the afferent fibres and the spinal circuitry are all exposed to an intrathecally applied drug.

**Figure 4:**
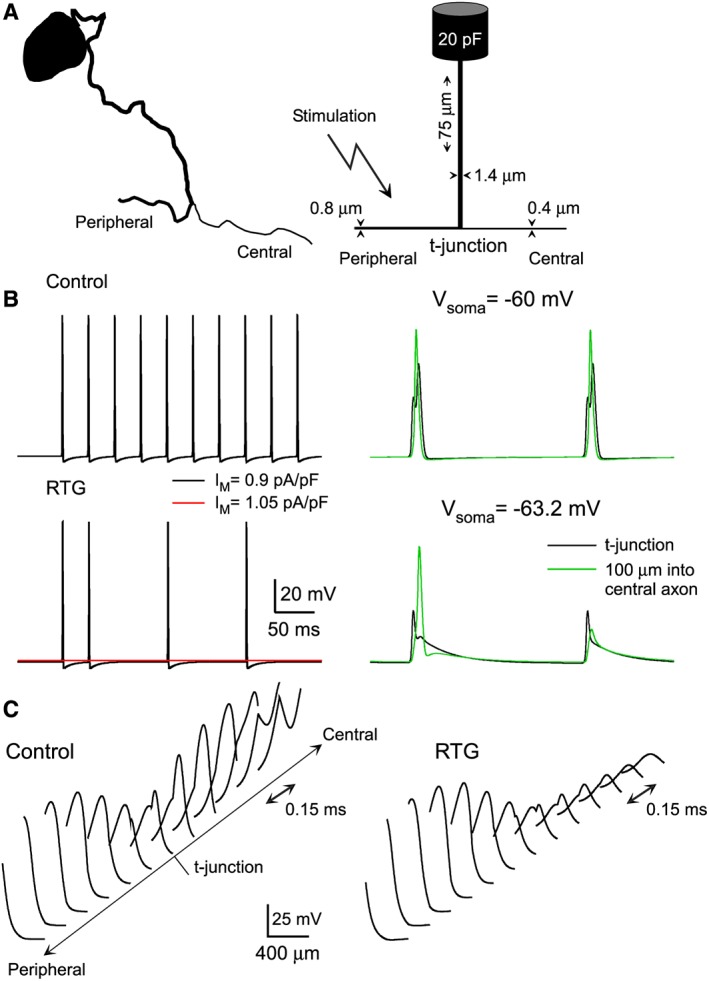
Biophysical model of a small‐diameter unmyelinated DRG neuron. (A) Shown on the left is a drawing of soma and stem axon of small‐diameter cat DRG neuron (based on micrograph from Ha, 1970); on the right are the morphological dimensions of the model neuron. (B) Enhancing the activity of M channels with virtual retigabine (−30 mV shift in activation curve, 1.5 fold increase in conductance density) hyperpolarized the t‐junction and reduced (at initial I_M_ density of 0.9 pA/pF) or abolished (at initial I_M_ density of 1.05 pA/pF) spike propagation in the model neuron. (C) Retigabine reduces the likelihood of spike generation in the central axon. Action potential amplitude is plotted as function of distance along 100 μm of peripheral and central axon sections with the t‐junction in the middle. In control conditions, spike amplitude decreases when approaching the t‐junction, due to the impedance load of the bifurcation. On the central side of the t‐junction, a delayed action potential develops and grows with distance from the t‐junction. When retigabine is added, the spike amplitude in the peripheral axon is not significantly affected; in contrast, the spike fails on the central side of the t‐junction and its decay is recorded (modified from Du *et al*., 2014, with permission).

Interestingly, a focal injection of flupirtine onto un‐injured sciatic nerve did not significantly affect noxious heat sensitivity of the plantar surface of the paw (Rose *et al.,* 2011), suggesting that the propagation of peripheral nociceptive signals through the axons is perhaps less affected by M channel activity, as compared to their initiation or transmission. However, there is one site within the axon of a somatosensory neuron where propagation of the peripherally‐derived action potentials can be effectively disrupted by M channel activation, and it is the point at which the axon, emanating from the cell body, bifurcates into the peripheral and central branches, the t‐junction (Figures 2 and 4A). The bifurcation of an afferent axon is a point of lowest safety factor for action potential propagation (Stoney, 1985; Luscher *et al.,* 1994; Debanne, 2004; Gemes *et al.,* 2013; Du *et al.,* 2014; Sundt *et al.,* 2015), and both experiments and computer modelling suggest that hyperpolarization of a t‐junction (e.g. with retigabine) can result in the failure of an action potential to propagate (Du *et al.,* 2014; Sundt *et al.,* 2015; Figure 4).

In order to better understand the filtering role of the t‐junction, we recently developed a computational model of a small‐diameter, unmyelinated mammalian DRG neuron (Du *et al.,* 2014; Sundt *et al.,* 2015; Figure 4) based on available anatomical data (Ha, 1970; Suh *et al.,* 1984; Hoheisel and Mense, 1987). The model revealed that due to the electrotonically short stem axon (Figure 4A), somatic/perisomatic hyperpolarization in combination with the increased membrane conductance, produced by ‘M channel’ potentiation (or by other similar manoeuvres), further lowered the safety factor at the t‐junction and, as a result, interfered with action potential propagation (Figure 4B, C). Without a t‐junction, the safety factor in the axon is relatively high, and comparable ion channel modulation, for example, in the central axon distal to the t‐junction, had no effect on spike transmission. The model explains well why an injection of flupirtine into the sciatic nerve did not significantly affect pain thresholds (Rose *et al.,* 2011). Whereas, as predicted by the model, focal infusion of retigabine directly on to the L5 DRG *in vivo* (*via* an implanted cannula) strongly alleviated pain produced by a hind paw injection of bradykinin (Du *et al.,* 2014).

In summary, current evidence suggests that the antinociceptive effect of M channel openers is mostly localized to peripheral nociceptive fibres, and within these, the action is most likely localized to the sites of action potential initiation (peripheral nerve endings, ectopic sites), propagation (the t‐junctions) and transmission (central terminals).

## Conclusions and future perspectives

The fifteen or so years since the first report of the presence of functional M channels in peripheral somatosensory neurons (Passmore *et al.,* 2003) has seen rapid progress in our understanding of the role these channels play in nociception. It is now firmly established that (i) the M current is an important control mechanism, which ‘clamps’ *E*_rest_ of nociceptors and sets their resting excitability parameters (e.g. threshold current); (ii) M channel openers consistently display analgesic efficacy in most experimental pain models; and (iii) both acute inhibition of M channel activity or long‐term down‐regulation of M channel abundance (e.g. *via* the epigenetic mechanisms) often drives nociceptors into an overexcitable state, which is associated with acute or chronic pain. M channels are therefore a validated drug target for the treatment of pain, and many pharmaceutical companies are investing in the development of new M channel openers. There are, however, a few issues that hamper the progress. Particularly, (i) there is still no clarity with regards to the M channel subunit expression profile in known subpopulations of sensory neurons and (ii) we are still short of the set of truly subunit‐selective M channel openers with good drug‐like properties. Both these shortcomings should be solved in order to be able to target M channels in crucial subpopulations of nociceptors more specifically.

Another consideration regarding possible refinement of current drug design strategies arises from the fact that while current therapeutic M channel openers are designed for the systemic application (i.e. oral), the analgesic efficacy of such drugs is most likely peripheral. It is also important to point out that the dorsal roots and the central terminals of the nociceptive fibres are protected by the blood–brain barrier (BBB) within the spinal cord, whereas the afferent axons within the peripheral nerves are protected by the blood‐nerve barrier (BNB). Yet spinal ganglia themselves have no such barrier and are exposed to the circulation to a significant extent (Devor, 1999). Since somatic/perisomatic M channels are capable of controlling action potentials propagating through the t‐junctions of nociceptors, it seems logical to propose that M channel openers with poor BBB/BNB permeability can still have analgesic efficacy due to their action within peripheral ganglia, yet the central side effects of such compounds could be significantly reduced. Future research will test if such a strategy can be implemented.

### Nomenclature of targets and ligands

Key protein targets and ligands in this article are hyperlinked to corresponding entries in http://www.guidetopharmacology.org, the common portal for data from the IUPHAR/BPS Guide to PHARMACOLOGY (Southan *et al*., 2016), and are permanently archived in the Concise Guide to PHARMACOLOGY 2015/16 (Alexander *et al.,* 2015a,b,c,d).

## Conflict of interest

The authors declare no conflicts of interest.
